# Association of Ozone Exposures with the risk of thyroid nodules in Hunan Province: a population-based cohort study

**DOI:** 10.1186/s12940-022-00874-8

**Published:** 2022-07-08

**Authors:** Qiao He, Min Wu, Qiman Shi, Hailong Tan, Bo Wei, Neng Tang, Jianjun Chen, Mian Liu, Saili Duan, Shi Chang, Peng Huang

**Affiliations:** 1 Department of General Surgery, Xiangya Hospital Central South University, Changsha, 410008 Hunan China; 2 College of Geomatics and Geoinformation, Guilin University of Technology, Guilin, 541006 Guangxi China; 3 Clinical Research Center for Thyroid Diseases in Hunan Province, Changsha, 410008 Hunan China; 4 Hunan Provincial Engineering Research Center for Thyroid and Related Diseases Treatment Technology, Changsha, 410008 Hunan China; 5 National Clinical Research Center for Geriatric Disorders (Xiangya Hospital), Changsha, 410008 Hunan China

**Keywords:** Thyroid nodules, Air pollution, Ozone exposure

## Abstract

**Background:**

Increasing evidence associates air pollution with thyroid dysfunction, whereas the potential relationship between exposure to ozone (O_3_) and Thyroid Nodules (TNs) is unclear.

**Methods:**

This retrospective cohort study investigated the association between O_3_ exposure and TNs in Hunan province, enrolling 191,357 Chinese adults who lived in Hunan province from January 2009 to December 2019 and received voluntary medical examinations. Individual exposure levels to O_3_ from 2010 to 2019 were measured on account of participants’ residential addresses at the district level. Associations of O_3_ exposure with the risk of incidental TNs were assessed by restricted cubic splines and surveyed as odds ratios after adjusting for demographic factors.

**Results:**

In total, 81,900 adults were newly diagnosed with TNs during the study period. Age-standardized TNs detection rate in Hunan province increased from 25.9 to 46.3% between 2010 and 2019, with the greatest annual percent change being 8.1 [95% CI, 7.3–8.8]. A similar trend has been found in all tumor sizes, ages, and both sexes. O_3_ exposure presented a statistically significant dose-dependent positive correlation (greater than 0.036 ppm) with TNs. Similarly, long-term exposure to high levels of O_3_ (1-year average O_3_ concentrations exceeding 0.0417 ppm) was found positively associated with increased TSH levels.

**Conclusions:**

High-level O_3_ exposure in the long term was associated with an increase in TSH. Consequently, increased TSH was related to the increased risk of TNs. Being exposed to high-level O_3_ in the long term was related to the increased detection rates of TNs in Hunan province, which could be mediated by TSH.

**Supplementary Information:**

The online version contains supplementary material available at 10.1186/s12940-022-00874-8.

## Background

Thyroid Nodules (TNs) present to be a kind of common clinical manifestation, occurring in 5% of the population, of which 7–15% turn out to be thyroid cancer [1]. In the United States, the morbidity of differentiated thyroid cancer tripled during the past 30 years [2]. Incremental evidence implicates so steady an increase that cannot be entirely ascribed to more sensitive diagnostic procedures and more intensive thyroid nodule screening [3], yet the reasons for the rapid increase of TNs remain elusive. Several environmental factors which show reliable candidates to stand for this increase have been observed, including improved iodine intake [4], exposure to various toxic compounds [5], nutritional deficiencies [6], eating habits, and comorbidities [7]. It has been reported that increased thyroid-stimulating hormone (TSH) or elevation within the normal range is relevant to an increased risk of malignancy in TNs [8]. Iodine deficiency, through regulating TSH levels, is a recognized risk factor for various thyroid diseases including TNs [4]. However, the fact that salt has been iodized all over China since 1996 has not stopped the incidence of thyroid cancer from markedly ascending, even in regions (including Hunan Province) with enough iodine intake [9, 10].

Air pollution is an unneglectable public health hazard [11]. Exposure to air pollutants, such as particulate matter (PM), Ozone (O_3_), nitrogen dioxide (NO_2_), and sulfur dioxide (SO_2_), has been proven to be related to public health and daily mortality worldwide [11, 12]. O_3_ is formed in the atmosphere through photochemical reactions, which lead to the action of ultraviolet light on precursor pollutants. A large body of literature links short-term O_3_ exposure to a wide range of cardiovascular diseases [13] and dry eye disease [14] but the relationship between long-term O_3_ exposure and some conditions is still poorly understood. Increasing evidence associates air pollution with thyroid dysfunction. Recent research has inspected the relationship between exposure to environmental and traffic-related air pollution and the thyroid function of pregnant women, which implied a strong link between ambient air pollution and thyroid dysfunction, both in pregnant women and newborns [15, 16]. Moreover, evidence indicates that short-term O_3_ exposure gives rise to a crucial decrease in circulating thyroid hormones and TSH in rats [17], as well as the threshold of goiter in several species of sharks [18]. Nevertheless, the correlation between ambient exposure to O_3_ and TNs has not been defined.

We hypothesized that high exposure to O_3_ is in connection with increased incidence of TNs and that abnormal thyroid function induced by O_3_ exposure may mediate and strengthen that association. To identify the associations between ambient exposure to O_3_ and TNs, we retrospectively analyzed 10 years of ambient O_3_ exposure in Hunan province as well as the age-adjusted TNs detection rate in 191,357 adults during the same period. We also examined and matched some risk factors for TNs, such as age, sex, BMI, triglycerides (TG), and total cholesterol (TC), which largely controlled potential confounding sociodemographic, behavioral, and clinical factors. This large sample study spanning ten years provides good insight into the effects of ambient O_3_ exposure on TNs.

## Methods

### Study population and data collection

We retrospectively collected voluntary medical examination records in the Health Management Center of Xiangya Hospital Central South University from January 2010 to December 2019. Xiangya Hospital Central South University is a top-ranked hospital with a good reputation in South-Central China, receiving patients from the whole Hunan province. Hunan Province is an area with rapid economic development, far from the ocean but with adequate iodine nutrition in South-Central China. We also obtained the measurement data of each air pollutant in every district.

This study was permitted by the Expert Committee of Xiangya Hospital Central South University in China (equivalent to an Institutional Review Board) and complied with the tenets of the Declaration of Helsinki. In this 10-year large research cohort, we recruited those (1) older than 18 years, (2) who were long-term residents (working and living more than 10 months per year) in Hunan province from January 2009 to December 2019, (3) who received voluntary medical examination including thyroid ultrasonography, (4) without a history of thyroid surgery or radiotherapy, (5) with no major chronic diseases as self-reported, (6) who had completed a questionnaire including demographic data and residential addresses before the physical examination.

**Fig. 1:**
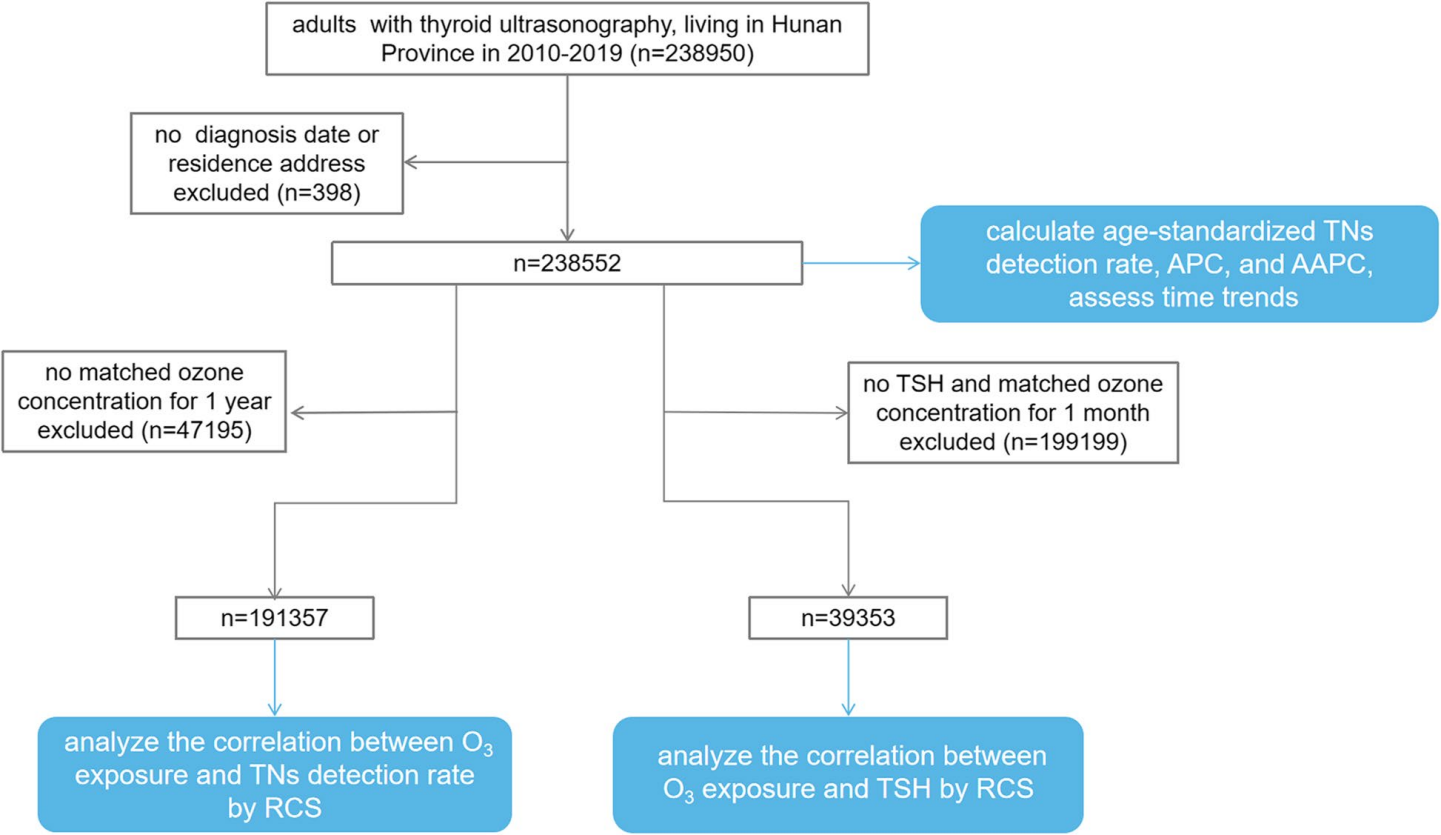
The flowchart for exclusion and analysis

TNs were diagnosed with high-resolution ultrasound and thyroid function was measured via serum concentrations of free T4, T3, and TSH. In the cases of repeated examinations, we used the last records for research. For those who were found with TNs in multiple physical examinations, we chose the first records with TNs diagnosis for research. Eventually, we analyzed the medical records of 191,357 subjects from 429,909 Chinese adults in the database. The flowchart for exclusion and analysis is shown in Fig. 1.

The residence location of a participant was determined by the questionnaire. Residential locations were described in detail to a district of the city and even the neighborhoods within the district. Thyroid nodule screening has become a routine item of physical examination, with low cost and comprehensive coverage of urban and rural areas.

### Air pollution exposures

Ambient air quality monitoring data from the China Air Quality Online Monitoring and Analysis Platform (https://www.aqistudy.cn/historydata/index.php) was used in this study. We downloaded O_3_ data (measured in ppm, parts per million) from 70 national ambient air quality monitoring stations in Hunan province. The distribution of national ambient air quality monitoring stations in each prefecture-level city is evenly covered. The district-level data used in our analysis is the average value obtained from actual measurements at each monitoring point in a given district.

### Covariates

Participants were asked to fill in questionnaires to provide basic information before the medical examination, including gender, age, height, weight, residential address, and chronic diseases. Because Thyroid nodule is a multifactorial disease, we regarded reported risk factors as covariates, including demographic factors (gender, age, regions) and obesity. Body mass index (BMI) was calculated as the weight in kilograms divided by the square of the height in meters (kg/m^2^). Overweight was defined as BMI ≥25 kg/m^2^ according to the World Health Organization (WHO-BMI) definition. Previous studies showed that thyroid function was significantly associated with dyslipidemia and seasonal changes [19, 20]. Therefore, we added TC, TG, and season as covariates when TSH was analyzed in the restricted cubic splines.

### Statistical analysis

This study applied R software (R 4.0.2) for data analysis, mainly using rms, ggplot2, and segmented packages. All tests were two-sided and *P*-values less than 0.05 deemed the corresponding result significant. For statistical description and comparison in the basic information, count data were indicated as frequencies (percentages), and the chi-square test was used for comparison between groups; those with normal distribution in the measurement data were indicated as mean (standard deviation, SD), and the student’s t-test was used for comparison between groups; those with non-normal distribution in the measurement data were indicated as median (interquartile range, IQR), and the Mann-Whitney U test was used for comparison among groups.

The crude detection rates of TNs were calculated in each stratum by age group (18–29, 30–39, 40–49, 50–59, 60–69, 70+ years old) and sex. Age-standardized rates by standard population were calculated using the age composition of the Hunan Province population according to the 6th census data released by the National Bureau of statistics. Mann-Kendall Trend Test was used to assess the changes in time series trends. We used the Joinpoint Regression Program (Version 4.9.0.0. March 2021; Statistical Research and Applications Branch, National Cancer Institute.) to get the APC (annual percent change) and AAPC (average annual percent change). Formulae are as shown (in 1–4). We employed a log-linear model {ln(*y*) = *β*_0_ + *β*_1_*x*} where y represents incidence, x represents the year of onset, and *β*_1_ is the regression coefficient. α is the statistical significance level (0.05). *APC*_*L*(*α*)_ *and APC*_*U*(*α*)_ are the lower and upper limit of the (1-α) confidence interval. d is the degree of freedom; s is the standard error of the regression coefficient (*β*_1_); t_d_^− 1^ (1-α/2) is the q percentile of the t distribution with the degree of freedom of d; *w*_*i*_ is the interval span width of each piecewise function (Number of years), and *β*_*i*_ is the regression coefficient corresponding to each interval.

1 $$APC=\frac{y_{x+1}-{y}_x}{y_x}=\left({e}^{\beta 1}-1\right)\times 100$$

2 $${APC}_{L\left(\alpha \right)}=100\left({e}^{\beta 1-s\times {t}_d^{-1}\left(1-\frac{\alpha }{2}\right)}-1\right)$$

3 $${APC}_{U\left(\alpha \right)}=100\left({e}^{\beta 1+s\times {t}_d^{-1}\left(1-\frac{\alpha }{2}\right)}-1\right)$$

4 $$AAPC=100\ \left({e}^{\sum {w}_i{\beta}_i/\sum {w}_i}-1\right)$$

Based on the age-standardized rates and the O_3_ exposure data, a geostatistical map was made to visually show the relationship between the geographical distribution of TNs detection rates and the annual mean ozone concentration of each prefecture. Spatial distribution of the annual age-standardized detection rate of TNs and average O_3_ concentration for each prefectural city were generated by ArcGIS 10.2 (http://www.esri.com/arcgis/about-arcgis). To further confirm this relationship, we detected this link through Spearman correlation by rank, excluding the outliers.

To evaluate the exposure-response relationship between O_3_ exposure and TNs detection rate, each case was matched with the O_3_ exposure concentration at the corresponding time and location. A total of 191,357 cases were included after excluding patients without matched O_3_ concentration data. The restricted cubic spline function fitted for Logistic Regression Model (R package rms) was employed, with age, gender, BMI, and prefecture controlled as confounding factors. The degree of freedom (df) was selected by evaluating the model fit according to the Akaike Information Criterion (AIC), and the preferred number of knots was 5, all equally spaced. The lowest point of exposure-response (O_3_ = 0.036 ppm) was used as the reference point (i.e., OR = 1) to plot the relationship curve between O_3_ and the OR of suffering from TNs. Furthermore, we used the same method to perform a 2-year O_3_ concentration analysis.

Similarly, each case was matched to the O_3_ exposure concentration at the corresponding time (1-month, 6-month, and 12-month) and location to explore the correlation between O_3_ exposure levels and TSH levels. Cases without thyroid function test results or unmatched O_3_ concentration data were excluded from the analysis. 39,353 valid data cases were included in the TSH and 1-month O_3_ exposure relationship analysis. The relationships between 1-month, 6-month, and 12-month average O_3_ exposure concentrations and TSH levels were also studied using restricted cubic spline functions fitted for generalized linear models based on Gaussian distribution, with gender, age, BMI, cholesterol, triglycerides, and season adjusted as confounders. The preferred number of equally spaced nodes of 3 was selected according to AIC. Subgroup analysis was performed by gender. Finally, the fitted curves were plotted. From the exposure-response curve, O_3_ concentrations (O_3_ = 0.0365 ppm, 0.0445 ppm, 0.0417 ppm) corresponding to the extreme points were obtained by iterating the generalized linear models in 0.0001-unit increments.

The relationship between thyroid function and TNs is already well established [4] and to verify this correlation, we compared the status of thyroid function (based on TSH, free T4 (FT4), and free T3 (FT3) levels) between the TN group and a control group. 39,353 adults were tested for thyroid function.

Finally, to further test the hypothesis, we also analyzed the relationship between O_3_ exposure, TNs, and TSH levels. The threshold was obtained from the curve of 1-year average O_3_ exposure and TNs. According to the threshold, the cases were divided into two groups (higher O_3_ group and lower O_3_ group). On the other hand, we obtained the TSH cut-off value by the ROC curve of TSH-TNs, determined by the largest Youden index. Similarly, we performed a TSH dichotomy (higher TSH group and lower TSH group). The Mann-Whitney U test and the Chi-Square test were applied to examine the continuous variables and the dichotomous variables of TSH in the higher O_3_ group, respectively. Further, the mediation analysis was performed by Model 4 of PROCESS (Version 4.1, Written by Andrew F. Hayes). The model included ozone exposure as an independent variable, thyroid nodules as a dependent variable, and TSH as a mediating variable, adjusted by sex, age, and BMI. We selected 5000 as the number of bootstrap samples for percentile bootstrap confidence intervals. Direct and indirect effects of ozone on TNs are on a log-odds metric.

## Results

The baseline characteristics of the study participants are shown in Table 1 and supplemental Table 1. The mean (interquartile range, IQR) age of the participants (95,316 men and 96,041 women) was 44 (34–53) years old. The mean (IQR) levels of TG, O_3_ (1-year average exposure), and O_3_ (2-year average exposure) during the study period were 1.32 (0.93–1.99) mmol/L, 0.0408 (0.0392–0.0418) ppm, and 0.0410 (0.0390–0.0415) ppm, respectively. The mean (SD) levels of BMI and TC were 23.60 (3.34) and 5.08 (1.02) mmol/L. The detection rate of newly diagnosed TNs was 42.8% (81,900/191357) and was significantly related to age, sex, BMI, overweight, TG, TC, and the exposure level of O_3_ (*P* < 0.001). TNs were more likely to occur in adults of older age, female sex, who had higher BMI, TG or TC levels, and higher levels of O_3_ exposure.

**Table 1:** Participant Characteristics of the study population

Parameters	Total (*n* = 191,357)	With detected TNs (*n* = 81,900)	Without thyroid nodule (*n* = 109,457)	*P* Value
Age, median (IQR), y	44 (34–53)	49 (39–58)	40 (31–50)	< .001
Sex				< .001
Men	95,316 (49.81)	34,231 (41.80)	61,085 (55.81)	
Women	96,041 (50.19)	47,669 (58.20)	48,372 (44.19)	
BMI, mean (SD)	23.60 (3.34)	23.76 (3.26)	23.48 (3.39)	< .001
Overweight				< .001
No	99,250 (51.87)	41,040 (50.11)	58,210 (53.18)	
Yes	75,478 (39.44)	33,327 (40.69)	42,151 (38.51)	
NA	16,629 (8.69)	7533 (9.20)	9096 (8.31)	
TG, median (IQR), mmol/L	1.32 (0.93–1.99)	1.35 (0.95–1.99)	1.30 (0.91–1.99)	< .001
TC, median (SD), mmol/L	5.08 (1.02)	5.14 (1.02)	5.04 (1.01)	< .001
O_3_ (1-year average exposure level), median (IQR), ppb	0.0408 (0.0392–0.0418)	0.0409 (0.0396–0.0419)	0.0407 (0.0391–0.0418)	< .001
O_3_ (2-year average exposure level), median (IQR), ppb	0.0410 (0.0390–0.0415)	0.4101 (0.3924–0.4157)	0.4092 (0.3892–0.4147)	< .001

*IQR* interquartile range, *BMI* Body mass index, *TG* triglycerides, *TC* total cholesterol, *O*_3_ Ozone

**Fig. 2:**
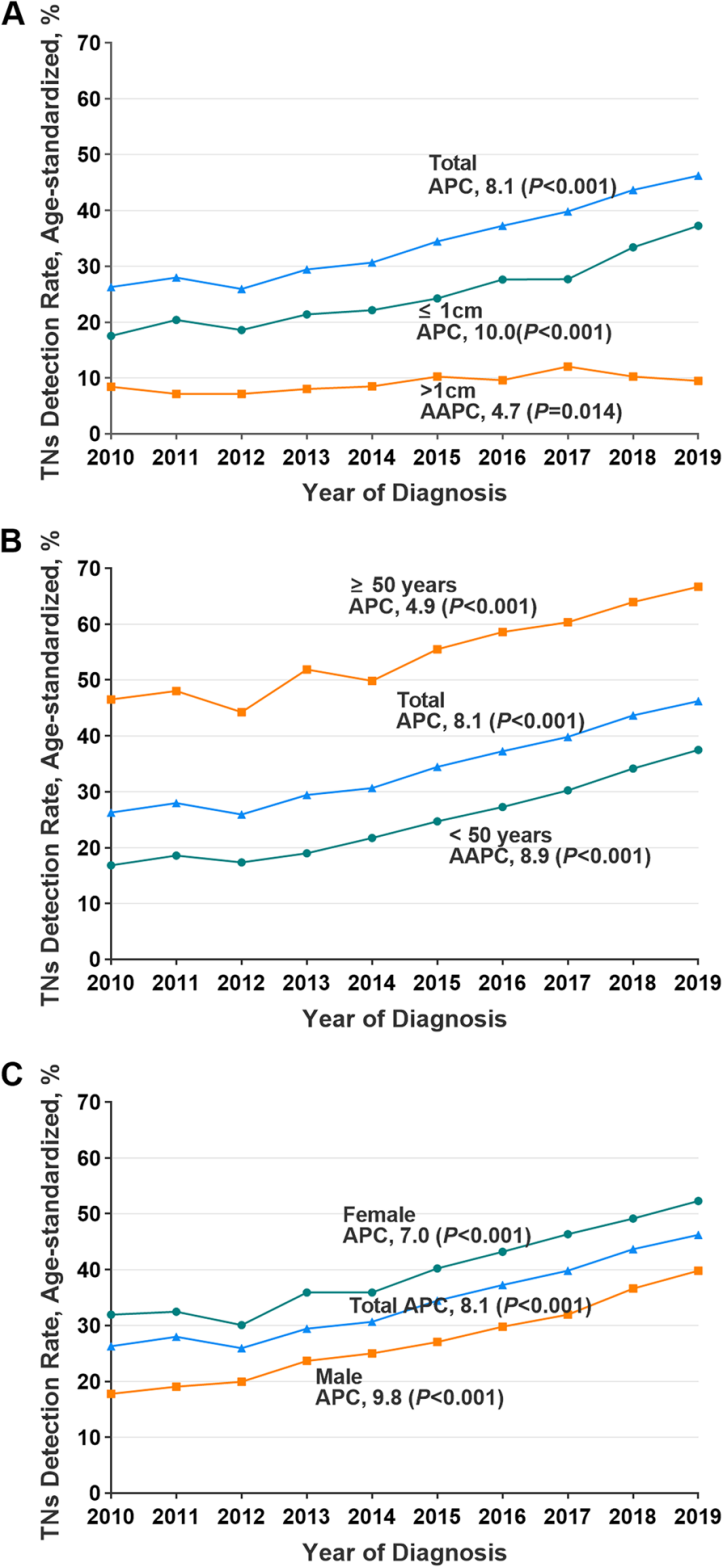
Trends in TNs Detection Rates, Stratified by Tumor Size, Age and Sex, 2010–2019

From 2010 to 2019, the age-standardized TNs detection rate in Hunan province significantly increased (from 25.9 to 46.3%, APC, 8.1 [95% CI, 7.3–8.8]; *P* < 0.001), with small nodule (≤1 cm, APC, 10.0 [95% CI, 8.2–11.9]; *P* < 0.001), male (APC, 9.8 [95% CI, 9.0–10.6]; *P* < 0.001) and youth (< 50 years, AAPC, 8.9 [95% CI, 5.2–12.8]; *P* < 0.001) preponderance. In addition, detection rates of TNs significantly rose in all tumor sizes, ages, and both sexes (Fig. 2, Supplemental Tables 2–4, and Supplemental Figs. 1–4).

**Fig. 3:**
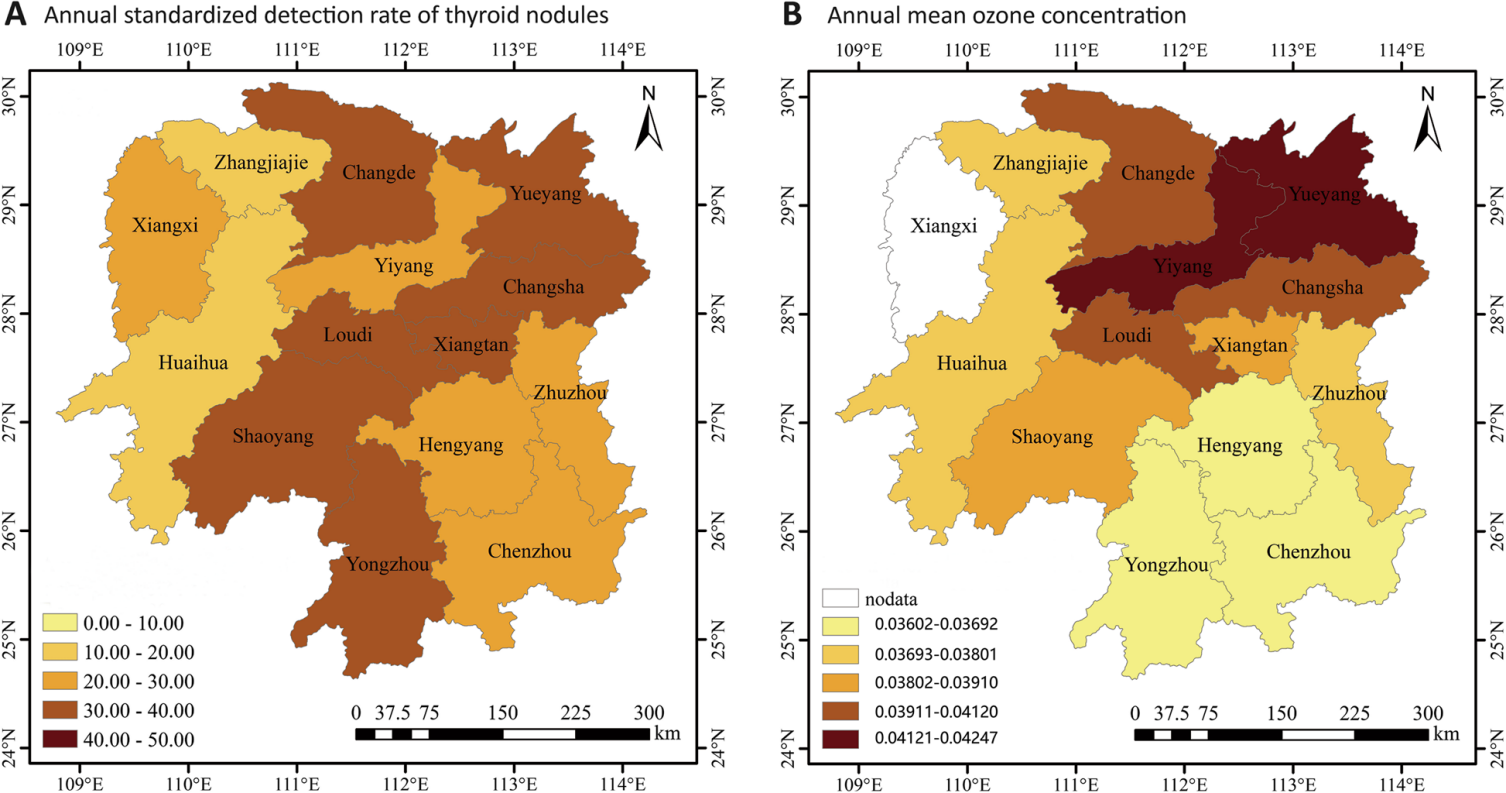
Spatial distribution of annual standardized detection rate of TNs and average O_3_ concentration for each prefectural city in Hunan province in Hunan province from 2010 to 2019

The grade distribution map showed that the yearly age-standardized detection rates of TNs of seven prefectural cities in Hunan province show a thyroid nodule detection rate of more than 40% (including Changde, Yueyang, Changsha, Loudi, Xiangtan, Shaoyang, Yongzhou), accounting for 50% of the total number of prefectural cities (Fig. 3). In addition, there is a spatial agglomeration of ambient O_3_ exposure in Hunan province according to the decade annual mean O_3_ concentration map, and the areas with the most severe pollution (O_3_ concentration of more than 0.412 ppm), such as Yueyang, Yiyang, Changde, Changsha, and Loudi are concentrated in the north-central areas of Hunan province, (Fig. 3B). The areas with high detection rates of TNs approximately overlap with the geographic regions where ozone exposure pollution is most severe. When all cities were included, the confirmatory ranking test revealed no significant connection (r_s_ = 0.209, *P* = 0.494). Nonetheless, when Yongzhou, Yiyang, and Xiangtan were eliminated as outliers, annual mean ozone concentrations of cities were significantly positively connected with TN detection rates (r_s_ = 0.64, *P* = 0.048). In other words, the TNs detection rates were closely related to the exposure levels of O_3_.

**Fig. 4:**
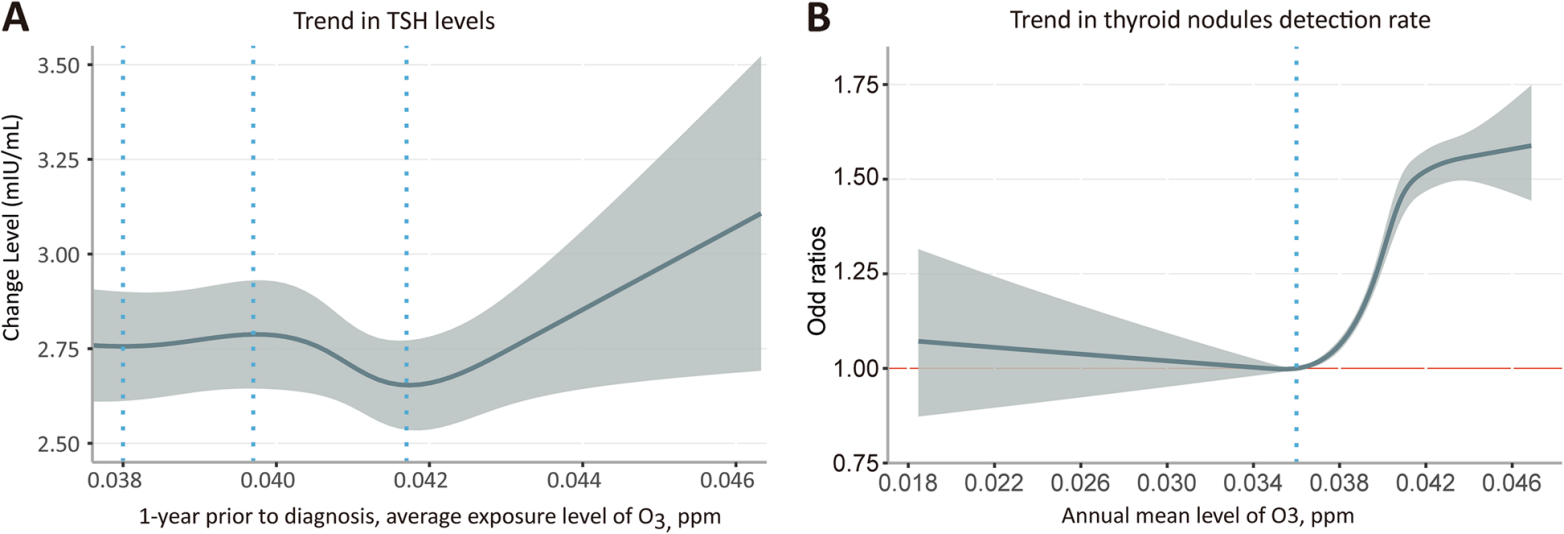
Multivariable Regression Models for TSH Level and TNs with Restricted Cubic Splines

Further analysis of the relationship between O_3_ exposure and TNs showed that the ORs for incident TNs based on O_3_ exposure exhibited a statistically significant dose-dependent elevation (when the O_3_ levels were greater than 0.036 ppm), and the association remained robust even after adjusting for age, sex, BMI, and location (Fig. 4B). It should be noted that the absolute OR value was greater than 1, when O_3_ exposure concentration was greater than 0.0408 ppm, suggesting that O_3_ could be a risk factor for TNs.

We observed a negative correlation between decreased TSH levels and short-term exposure to O_3_, such as 1-month average O_3_ concentrations exceeding 0.0365 ppm before diagnosis or 6-month average O_3_ concentrations exceeding 0.0445 ppm (supplemental Fig. 5A, B). Nonetheless, 1-year average O_3_ concentrations exceeding 0.0417 ppm were found positively associated with increased TSH levels (Fig. 4A). Accordingly, exposure to O_3_ in the long term was associated with an increase in TSH levels.

Of the 39,353 adults that were tested for thyroid function, 17,265 were diagnosed with TNs, and 22,088 were not. The results of our verification of the correlation between thyroid function and TNs showed that, although the results of almost all the thyroid functions were within the normal range, the mean (IQR) levels of TSH in individuals with TNs were prominently higher than in those without TNs (*P* = 0.007, 2.21 (1.47–3.3) vs. 2.19 (1.49–3.16)), and the mean (IQR) levels of FT3 levels in adults with TNs were observably lower than in those without TNs (*P* < 0.001, 4.52 (4.10–5.00) vs. 4.65 (4.18–5.15)), but no significant difference was found in FT4 levels between the two groups (Supplemental Table 1).

Further analyses of the relationship between O_3_ exposure, TSH level, and TNs are fully shown in Table 2. The TSH cut-off value was 3.2 mIU/L, obtained from the ROC curve of TSH-TNs. Based on the threshold values of O_3_ and TSH, all 191,357 Chinese adults were divided into two groups, respectively. The detection rates of TNs were significantly higher in the higher O_3_ group (*P* < 0.001) and the higher TSH group (*P* < 0.001). Meanwhile, TSH levels of adults with TNs were prominently higher than those without TNs in the higher O_3_ group (*P* = 0.006, 2.21 (1.47–3.3) vs. 2.18 (1.48–3.14)), which did not exist in the lower O_3_ group (*P* = 0.245). In the higher O_3_ group, the TNs detection rate of the higher TSH group was significantly higher than that of the lower TSH group (*P* < 0.001). The mediation analysis showed that the direct effect of ozone exposure on the dependent variable TN was 42.078 (33.516–50.639), accounting for 99.35% of the total effect. The indirect effect of ozone exposure on TNs through TSH level was 0.277 (0.0424–0.656), accounting for 0.65% of the total effect.

**Table 2:** The relationship between O3 exposure, TSH level, and TNs

Parameters	Total	With TNs	Without TNs	*P* Value
O_3_ exposure classification				
Higher O_3_ group^a^	94,695	42,671(45,1)	52,024(54.9)	< .001
Lower O_3_ group^b^	96,662	39,230(40.6)	57,432(59.4)	
TSH classification				
Higher TSH group^c^	9689	4516	5173	< .001
Lower TSH group^d^	28,392	12,276	16,116	
Higher O_3_ group				
With TSH data	18,214	9034	9180	
TSH, median (IQR)		2.21(1.47–3.30)	2.18(1.48–3.14)	0.006
Higher TSH group	4658	2448	2210	< .001
Lower TSH group	13,556	6586	6970	
Lower O_3_ group				
With TSH data	19,867	7758	12,109	
TSH, median (IQR)		2.21(1.47–3.3)	2.20(1.50–3.17)	0.245

Higher group^a^, 1-year average O_3_ concentrations above the threshold (0.0408 ppm); Lower group^b^, 1-year average O_3_ concentrations below the threshold; Higher TSH group^c^, TSH level above the threshold (3.2 mIU/L); Lower TSH group^d^, TSH level below the threshold

## Discussion

Employing a cohort study, we examined the relationship between a time-varying 10-year mean O_3_ exposure and incidental TNs in Hunan province, an area with rapid economic development and adequate iodine nutrition in South-Central China. We found a statistically significant increase in TNs detection rates, which may be attributed to long-term, high-level O_3_ exposure mediated by TSH.

In recent decades, the prevalence of thyroid nodules (TNs) has steadily increased [21]. As people’s living standards have improved as a result of rapid economic growth, it has become easier for them to obtain medical examinations, thereby increasing the detection rate of TNs; more sensitive diagnostic procedures also have increased the detection of TNs by allowing the detection of smaller nodules [3]. However, incremental evidence implicates so steady an increase that cannot be entirely ascribed to more intensive thyroid nodule screening and more sensitive diagnostic procedures [3]. Several environmental factors which could lead to the increase have been observed, including iodine intake [4], toxic compounds [22], nutrition [6], obesity [23], and so on. Iodine is a trace element required for thyroid hormone synthesis and is an essential component of the thyroid cells’ functional microenvironment. Both iodine deficiency and excessive iodine may result in thyroid diseases such as thyroid nodules, hypothyroidism, and autoimmune diseases [4, 24–26]. As a country suffering from mild to moderate iodine deficiency, China implemented Universal Salt Iodization (USI) in 1996. By 2010, 28 provinces (including Hunan Province) had realized the goal of eliminating iodine-deficient disorders [27]. During the two decades of USI, the Chinese population has been consecutively exposed to three different stages of iodine nutritional status: excessive iodine intake (median urine iodine concentration, UIC ≥300 μg/L) from 1996 to 2001, more than adequate iodine intake (median UIC 200–299 μg/L) from 2002 to 2011, and adequate iodine intake (median UIC 100–199 μg/L) from 2012 to 2016 [27]. Iodine prophylaxis has played a key role in alleviating goiter and other iodine deficiency disorders and modulated the composition of thyroid disease [27]. It was reported that excessive iodine intake is positively associated with the risk of TNs [25, 26]. From the perspective of iodine nutrition level, excessive iodine intake may not be enough to explain the increase in TNs incidence, considering that the province’s iodine nutrition was not in an excessive iodine intake state during the period of this study. In our study, we found that long-term exposure to high levels of O_3_ would increase the risk of TNs.

To our knowledge, the present study is the first one investigating the correlation between the risk of incidental TNs and O_3_ concentration, which ultimately revealed an association between those two. Well-established evidence implied higher serum TSH concentrations would increase the likelihood of TNs and thyroid cancer [4]. Previously, four observational studies [15, 16, 28, 29] had particularly examined the relevance between air pollutants and thyroid function alteration of pregnant women and their newborns, which unveiled that PM2.5 and PM10 but not O_3_ were associated with the abnormal thyroid function, yet those results only explored the association at a short-term level. Of note, our findings confirmed that the relationship between O_3_ exposure and TSH level alteration was time-varying, namely short-term O_3_ exposure (1-month or 6-month) at high concentrations leads to the decline of TSH levels. In contrast, long-term exposure (1-year) to high levels of O_3_ leads to a rise in TSH levels. To date, the relationship between short-term O_3_ exposure and thyroid function has been presented in several studies [17, 30–33]. Consistent with our study, short-term O_3_ exposure has been associated with decreases in circulating TSH [17, 30–33]. Short-term O_3_ exposure was found to result in a remarkable reduction of circulating thyroid hormones and TSH in rats, which was interpreted as a possible adaptive mechanism to enhance the survival of rats during ozone exposure [17]. Huffman has observed the exacerbation of ozone effects in animals with hyperthyroidism [32]. Short-term O_3_ exposure has been reported to increase the levels of adrenal-derived circulating stress hormones and markedly decrease circulating TSH levels, indicating that short-term ozone exposure may function through the neuroendocrine axis, mediated by activation of central stress-response mechanisms [30, 34]. What’s more, this study was the first to establish the change of TSH through long-term O_3_ exposure (1-year) at high engagement. Perhaps, long-term ozone exposure induces thyroid inflammation, in which thyroid cells are destroyed. High TSH levels are required to promote the destruction-repair cycle of thyroid follicular epithelium cells to maintain thyroid homeostasis.

We agree with previous reports that increased TSH or elevation within the normal range is related to an increased risk of TNs. Our results show that long-term high ozone exposure is not only associated with elevated TSH but is also positively associated with thyroid nodule risk and this may be explained by higher serum TSH concentrations favoring the likelihood of TNs. The mediation analysis, together with the results of subgroup analyses, supports the idea that high ozone exposure, may play at least a partial role in thyroid nodule risk with TSH as a mediator. Considering these observations with our findings, we hypothesize that abnormal thyroid function can mediate the association between ozone exposure and TN incidence. However, multi-center studies and in-depth mechanistic research are required to confirm this.

Though our survey results revealed a long-term correlation between O_3_ exposure and TNs, the mechanism of that is still far from being fully understood and awaits further investigation. Hypothetically, the reaction of oxidative stress and remodeling of the microenvironment are tending to be the potential mechanisms since autoimmune processes may work on thyroid function for a more extended period [35]. Meanwhile, growing incidence implied that elevated TSH levels and thyroid autoimmunity were summarized as independent risk factors for thyroid cancer [36]. Such mechanisms have been identified in the pathological development of Hashimoto’s thyroiditis, which involved a shift from hyperthyroidism to hypothyroidism [37, 38]. Moreover, Hashimoto’s thyroiditis is an independent risk factor for incidental thyroid cancer [27].

Another explanation may lay in the long-term O_3_ exposure-induced Oxidative DNA damage. It is well-proven that the reactive oxygen species (ROS) that evade cellular defenses result in permanent or temporary damage to lipids, proteins, and nucleic acids [39]. Potassium bromate is a by-product of ozonation of high-bromide surface water to produce drinking water, as well as a rodent carcinogen that can produce tumors, including thyroid cancer [40, 41]. It has been established that potassium bromate renal carcinogenesis involves premutagenic base damage in DNA specially removed by base excision repair [42]. More direct evidence of the fact is a report describing the outbreak of goiter in a few species of sharks following the addition of ozone to a touch pool [18]. Four brown-banded bamboo sharks (*Chiloscyllium punctatum*) and 11 white-spotted bamboo sharks (*Chiloscyllium plagiosum*) housed in an aquarium with a high-level O_3_ exposure were diagnosed with hypothyroidism and multinodular goiter [18]. Yet, there is a lack of evidence that the development of TNs requires a latency period following exposure to O_3_, and further research is needed to access the optimal time lag between O_3_ exposure and the development of TNs.

As far as we know, our study is the first to explore the association between ozone exposure and thyroid nodules, and the first to verify the association between long-term ozone exposure and TSH, with a sufficiently large sample size but it inevitably has some limitations. The most obvious limitation is that the assessment of ozone exposure was not very accurate due to limited monitoring sites and the inability to apply a hybrid geophysical-statistical model. Due to some special factors, we can only obtain limited data from the public data of monitoring stations which may be clustered. In addition, the results of this study only partially reflect the detection rate of TNs in different regions of Hunan Province since the analysis depended on voluntary examination records and did not account for those who did not participate. We did not include diet (iodine intake) as a covariate in our analysis due to the lack of detailed data, instead, we assumed that iodine intake levels are balanced between groups since the iodine nutritional status of the Chinese population, in the period of this study, was almost in an adequate iodine intake state. Also, as the shapes of cities in Hunan province, such as Yiyang and Huaihua city, are irregular, even long and narrow, they may be closer to monitoring stations in nearby cities, creating the potential of producing inaccurate data.

## Conclusions

High-level O_3_ exposure in the long term was associated with an increase in TSH. Consequently, increased TSH was related to the increased risk of TNs. Being exposed to high-level O_3_ in the long term was related to the increased detection rates of TNs in Hunan province, which could be mediated by TSH.

## Supplementary Information

**Additional file 1: Supplemental Table 1.** Basic characteristics of 39,353 patients with thyroid function. **Supplemental Table 2.** Age-standardized detection rates of TNs in Hunan Province from 2010 to 2019, stratified by tumor size, all age, both sexes. **Supplemental Table 3.** Age-standardized detection rates of TNs in Hunan Province from 2010 to 2019, stratified by age, all tumor sizes, both sexes. **Supplemental Table 4.** Age-standardized detection rates of TNs in Hunan Province from 2010 to 2019, stratified by sex, all tumor sizes, all ages. **Supplemental Figure 1.** Trends in TNs Detection Rates, Stratified by Sex, all ages, 2010–2019. **Supplemental Figure 2.** Trends in TNs Detection Rates, Stratified by age, all tumor sizes, 2010–2019. **Supplemental Figure 3.** Trends in Detection Rates of TNs with the maximum diameter > 1 cm, Stratified by age, 2010–2019. **Supplemental Figure 4.** Trends in Detection Rates of TNs with the maximum diameter ≤ 1 cm, Stratified by age, 2010–2019. **Supplemental Figure 5.** Multivariable Regression Models for TSH Level with Restricted Cubic Splines.

## Abbreviations

TNs: Thyroid Nodules
O_3_: Ozone
TSH: Thyroid-stimulating hormone
PM: Particulate matter
NO_2_: Nitrogen dioxide
SO2: Sulfur dioxide
TG: Triglycerides
TC: Total cholesterol
BMI: Body mass index
WHO: World Health Organization
SD: Standard deviation
IQR: Interquartile range
FT4: Free thyroxine
FT3: Free triiodothyronine
APC: Annual percentage Change
AAPC: Average annual percentage change
AIC: Akaike information criterion
ppm: Parts per million
ROS: Reactive oxygen species
