# Supplementary material for: Association of Ozone Exposures with the risk of thyroid nodules in Hunan Province: a population-based cohort study
Source: Environ Health. 2022 Jul 8;21:65. doi: 10.1186/s12940-022-00874-8 (PMC9264600; doi:10.1186/s12940-022-00874-8)

**Supplemental materials**

**Association of Ozone Exposures with the Risk of Thyroid Nodules in Central Southern China:** **a population-based cohort study**

Qiao He^a^, Min Wu^a^, Qiman Shi^a^, Hailong Tan^a^, Bo Wei^a^, Neng Tang^a^, Jianjun Chen^b^, Mian Liu^a^, Saili Duan^a^, Shi Chang^a,c,d,e^, Peng Huang^a*^

*^a^ Department of General Surgery, Xiangya Hospital Central South University, Changsha 410008, Hunan, China*

*^b^ College of Geomatics and Geoinformation, Guilin University of Technology, Guilin 541006, Guangxi, China*

*^c^ Clinical Research Center for Thyroid Disease In Hunan Province, Changsha 410008, Hunan, China*

*^d^ Hunan Provincial Engineering Research Center for Thyroid and Related Diseases Treatment Technology, Changsha 410008, Hunan, China*

*^e^ National Clinical Research Center for Geriatric Disorders (Xiangya Hospital), Changsha 410008, Hunan, China*

**Corresponding Author: Peng Huang, E-mail address: xiangyahp@csu.edu.cn*

| Supplemental Table 1. Basic characteristics of 39353 patients with thyroid function | | | | |
| --- | --- | --- | --- | --- |
| Parameters | Total  (n = 39353) | With TNs  (n = 17265) | Without TNs  (n = 22088) | *P* Value |
| Age, median (IQR), y | 39 (30-51) | 46 (34-59) | 34 (29-45) | < .001^§^ |
| Sex |  |  |  | < .001^＊^ |
| Men | 12154 (30.89) | 4655 (26.96) | 7499 (33.95) |  |
| Women | 27199 (69.11) | 12610 (73.04) | 14589 (66.05) |  |
| BMI, mean (SD) | 22.77 (3.28) | 23.08 (3.23) | 22.53 (3.30) | < .001^＆^ |
| TSH, median (IQR), mIU/L | 2.20 (1.48-3.22) | 2.21 (1.47-3.3) | 2.19 (1.49-3.16) | < .001^#^ |
| Free T4, median (IQR), ng/dL | 15.03 (13.0-17.08) | 15.05 (13.03-17.05) | 15.02 (12.97-17.11) | .485^#^ |
| Free T3, median (IQR), ng/dL | 4.59 (3.14-5.09) | 4.52 (4.10-5.00) | 4.65 (4.18-5.15) | .14^#^ |
| TG, median (IQR), mmol/L | 1.16 (0.82-1.75) | 1.23 (0.87-1.84) | 1.11 (0.79-1.66) | < .001^§^ |
| TC, median (SD), mmol/L | 5.00 (0.98) | 5.08 (1.00) | 4.94 (0.96) | < .001^＆^ |
| O_3_ (1-month average exposure level), median (IQR), ppb | 0.0387 (0.0298-0.0541) | 0.047 (0.032-0.056) | 0.035 (0.028-0.052) | < .001^§^ |
| O_3_ (3-month average exposure level), median (IQR), ppb | 0.0396 (0.0243-0.0527) | 0.045 (0.026-0.053) | 0.030 (0.024-0.051) | < .001^§^ |
| O_3_ (6-month average exposure level), median (IQR), ppb | 0.0374 (0.0328-0.0473) | 0.041 (0.034-0.048) | 0.036 (0.032-0.046) | < .001^§^ |
| O_3_ (1-year average exposure level), median (IQR), ppb | 0.0406 (0.0380-0.0414) | 0.041 (0.039-0.042) | 0.040 (0.038-0.041) | < .001^§^ |
| IQR, interquartile range; BMI, Body mass index; TSH, thyrotropin; T4, thyroxine; TG, triglycerides; TC, total cholesterol; O_3_, Ozone  ^§^, Mann-Whitney U test; ^＊^, Chi-square test; ^＆^, student’s t-test; ^*^Binary logistic regression adjusted for age, sex, BMI, TG, and TC. | | | | |

Supplemental Table 2. Age-standardized detection rates of TNs in Hunan Province from 2010 to 2019, stratified by tumor size, all age, both sexes

| Year | ≤ 1 cm | | | > 1 cm | | Total | |
| --- | --- | --- | --- | --- | --- | --- | --- |
|  | Crude (%) | | Standard (%) | Crude (%) | Standard (%) | Crude (%) | Standard (%) |
| 2010 | 18.3 | | 17.5 | 9.2 | 8.5 | 27.7 | 26.3 |
| 2011 | 22.3 | | 20.4 | 8.4 | 7.1 | 31.2 | 28.0 |
| 2012 | 19.2 | | 18.6 | 7.0 | 7.2 | 26.4 | 25.9 |
| 2013 | 22.9 | | 21.4 | 8.6 | 8.0 | 31.4 | 29.5 |
| 2014 | 22.3 | | 22.1 | 8.5 | 8.5 | 30.8 | 30.7 |
| 2015 | 24.5 | | 24.2 | 10.4 | 10.2 | 34.9 | 34.5 |
| 2016 | 28.4 | | 27.6 | 10.1 | 9.6 | 38.5 | 37.2 |
| 2017 | 27.8 | | 27.7 | 12.5 | 12.1 | 40.4 | 39.8 |
| 2018 | 34.0 | | 33.4 | 10.4 | 10.2 | 44.5 | 43.6 |
| 2019 | 37.9 | | 37.2 | 10.1 | 9.5 | 48.1 | 46.3 |
| Mean | 30.1 | | 29.6 | 10.3 | 9.9 | 40.4 | 39.5 |
| Annual percentage change (APC) analysis | | | | | | | |
| APC | |  | 10.0 |  | 4.7 |  | 8.1 |
| Lower 95% CI | |  | 8.2 |  | 0.9 |  | 7.3 |
| Upper 95% CI | |  | 11.9 |  | 8.5 |  | 8.8 |
| *P* Value | |  | < .001 |  | .014 |  | < .001 |
| Mann-Kendall Trend Test | | | | | | | |
| z-stat | |  | 3.7566 |  | 2.1466 |  | 3.5777 |
| *P* Value | |  | .0001722 |  | .03182 |  | .0003466 |

**Supplemental Table 3. Age-standardized detection rates of TNs in Hunan Province from 2010 to 2019, stratified by age, all tumor sizes, both sexes**

| Year | ＜50 | | | ≥50 | | Total | |
| --- | --- | --- | --- | --- | --- | --- | --- |
|  | Crude (%) | | Standard (%) | Crude (%) | Standard (%) | Crude (%) | Standard (%) |
| 2010 | 20.4 | | 16.8 | 44.0 | 46.5 | 27.7 | 26.3 |
| 2011 | 21.8 | | 18.6 | 46.9 | 48.1 | 31.2 | 28.0 |
| 2012 | 20.3 | | 17.3 | 41.6 | 44.3 | 26.4 | 25.9 |
| 2013 | 22.3 | | 19.0 | 49.2 | 51.9 | 31.4 | 29.5 |
| 2014 | 23.1 | | 21.7 | 46.4 | 49.9 | 30.8 | 30.7 |
| 2015 | 26.3 | | 24.7 | 51.9 | 55.5 | 34.9 | 34.5 |
| 2016 | 29.2 | | 27.3 | 55.5 | 58.6 | 38.5 | 37.2 |
| 2017 | 31.9 | | 30.2 | 56.8 | 60.4 | 40.4 | 39.8 |
| 2018 | 35.5 | | 34.2 | 60.7 | 64.0 | 44.5 | 43.6 |
| 2019 | 38.0 | | 37.5 | 64.6 | 66.7 | 48.1 | 46.3 |
| Mean | 31.3 | | 29.8 | 57.3 | 60.2 | 40.4 | 39.5 |
| Annual percentage change (APC) analysis | | | | | | | |
| APC | |  | 11.6 |  | 4.9 |  | 8.1 |
| Lower 95% CI | |  | 10.9 |  | 4.1 |  | 7.3 |
| Upper 95% CI | |  | 12.3 |  | 5.8 |  | 8.8 |
| *P* Value | |  | < .001 |  | < .001 |  | < .001 |
| Mann-Kendall Trend Test | | | | | | | |
| z-stat | |  | 3.7566 |  | 3.3988 |  | 3.5777 |
| *P* Value | |  | .0001722 |  | .0006768 |  | .0003466 |

**Supplemental Table 4. Age-standardized detection rates of TNs in Hunan Province from 2010 to 2019, stratified by sex, all tumor sizes, all ages**

| Year | Female | | | Male | | Total | |
| --- | --- | --- | --- | --- | --- | --- | --- |
|  | Crude (%) | | Standard (%) | Crude (%) | Standard (%) | Crude (%) | Standard (%) |
| 2010 | 34.6 | | 32.8 | 18.9 | 17.7 | 27.7 | 26.3 |
| 2011 | 37.6 | | 33.4 | 20.9 | 19.0 | 31.2 | 28.0 |
| 2012 | 31.2 | | 30.9 | 20.9 | 19.9 | 26.3 | 26.0 |
| 2013 | 40.7 | | 36.9 | 24.4 | 23.6 | 34.4 | 29.4 |
| 2014 | 35.2 | | 36.9 | 35.2 | 25.0 | 30.8 | 30.7 |
| 2015 | 40.9 | | 41.4 | 26.6 | 27.0 | 34.9 | 34.5 |
| 2016 | 45.2 | | 44.4 | 28.4 | 29.8 | 38.6 | 37.2 |
| 2017 | 48.0 | | 47.7 | 31.7 | 31.9 | 40.4 | 39.8 |
| 2018 | 51.4 | | 50.5 | 37.5 | 36.6 | 44.5 | 43.6 |
| 2019 | 55.0 | | 53.8 | 41.3 | 39.8 | 48.1 | 46.8 |
| Mean | 47.2 | | 46.4 | 33.7 | 32.7 | 40.4 | 39.5 |
| Annual percentage change (APC) analysis | | | | | | | |
| APC | |  | 7.0 |  | 9.8 |  | 8.1 |
| Lower 95% CI | |  | 6.1 |  | 9.0 |  | 7.3 |
| Upper 95% CI | |  | 7.9 |  | 10.6 |  | 8.8 |
| *P* Value | |  | < .001 |  | < .001 |  | < .001 |
| Mann-Kendall Trend Test | | | | | | | |
| z-stat | |  | 3.5777 |  | 3.9355 |  | 3.5777 |
| *P* Value | |  | .0003466 |  | .00008303 |  | .0003466 |

Supplemental Figure 1. Trends in TNs Detection Rates, Stratified by Sex, all ages, 2010-2019
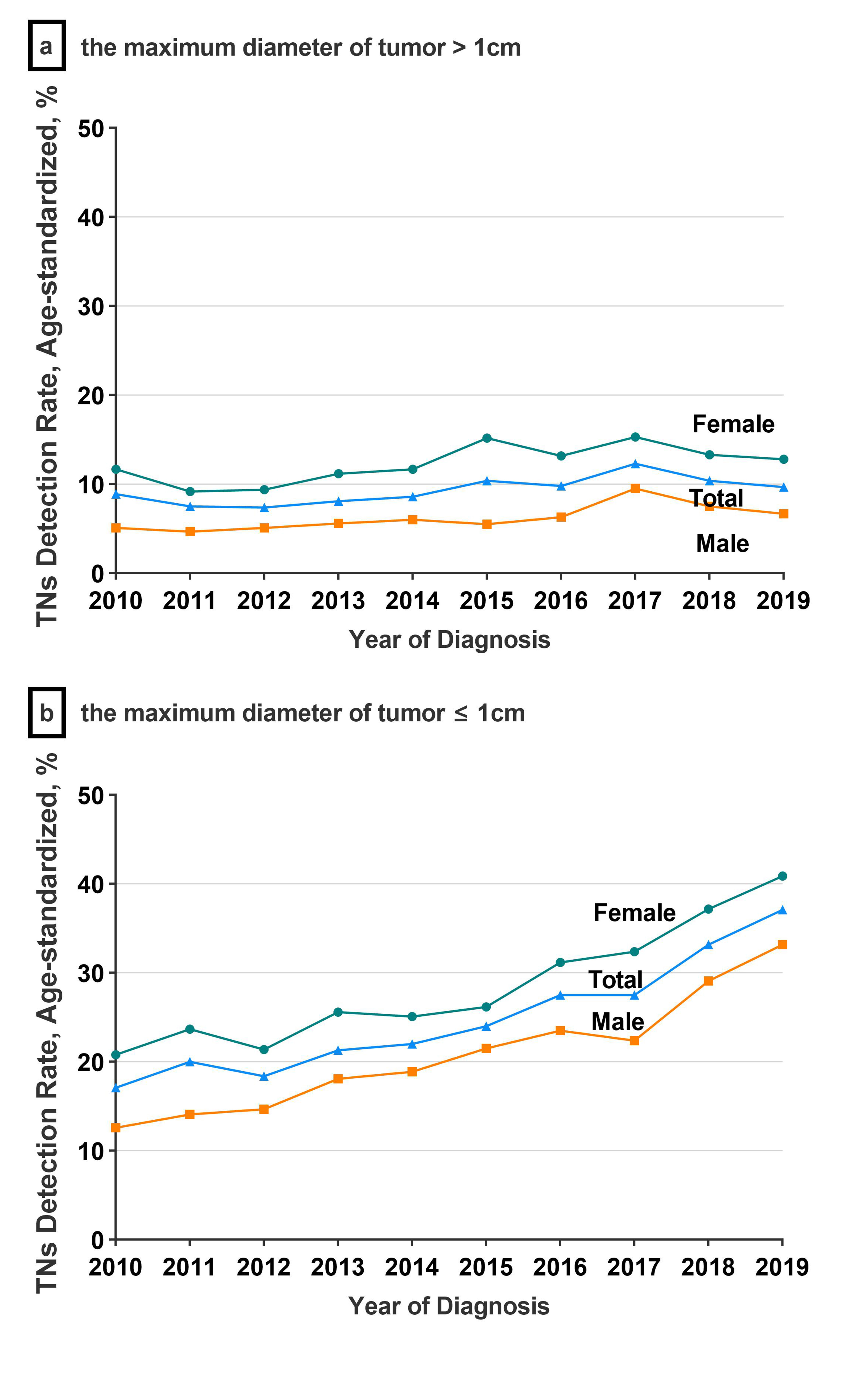


Supplemental Figure 2. Trends in TNs Detection Rates, Stratified by age, all tumor sizes, 2010-2019

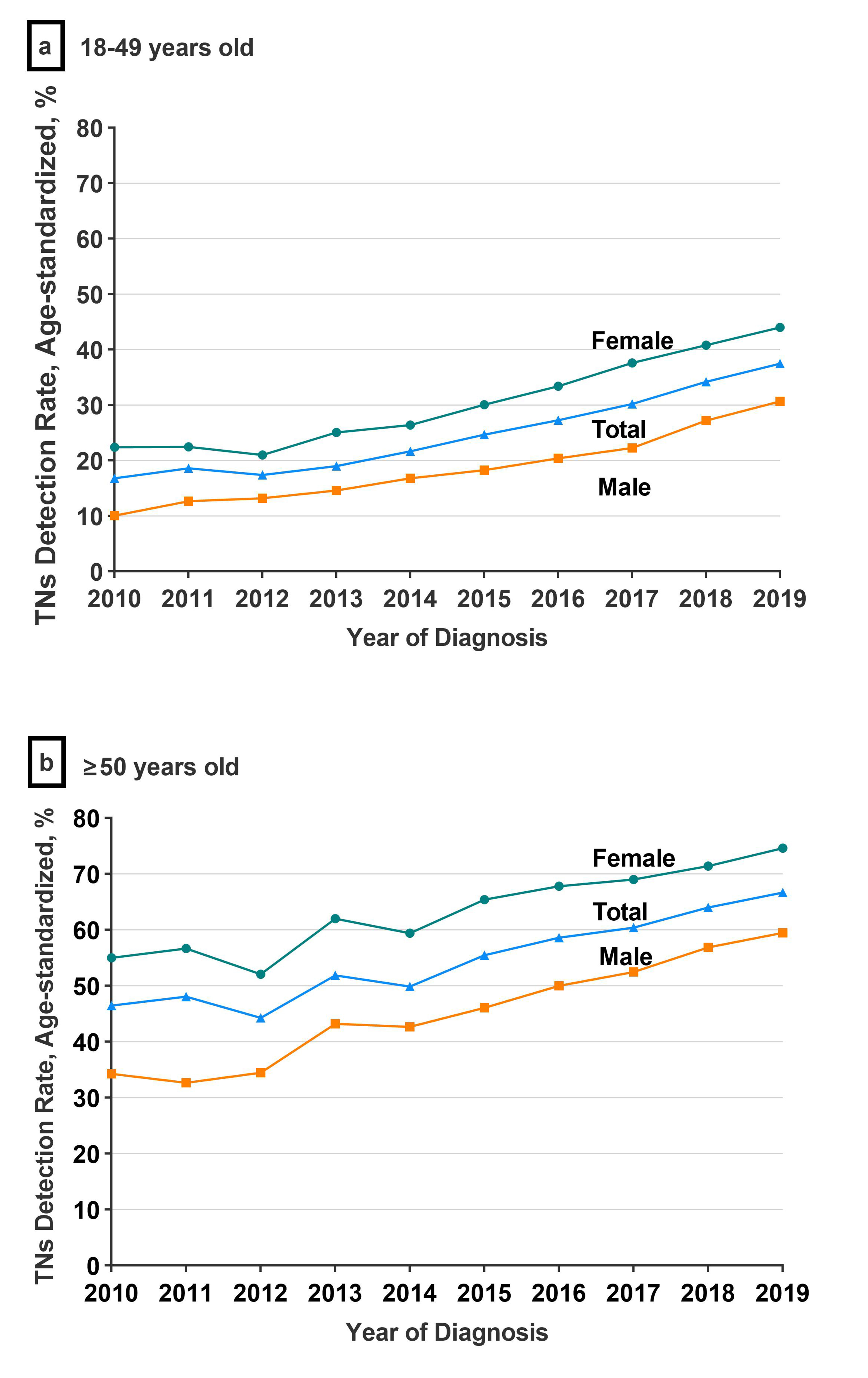


Supplemental Figure 3. Trends in Detection Rates of TNs with the maximum diameter >1 cm, Stratified by age, 2010-2019


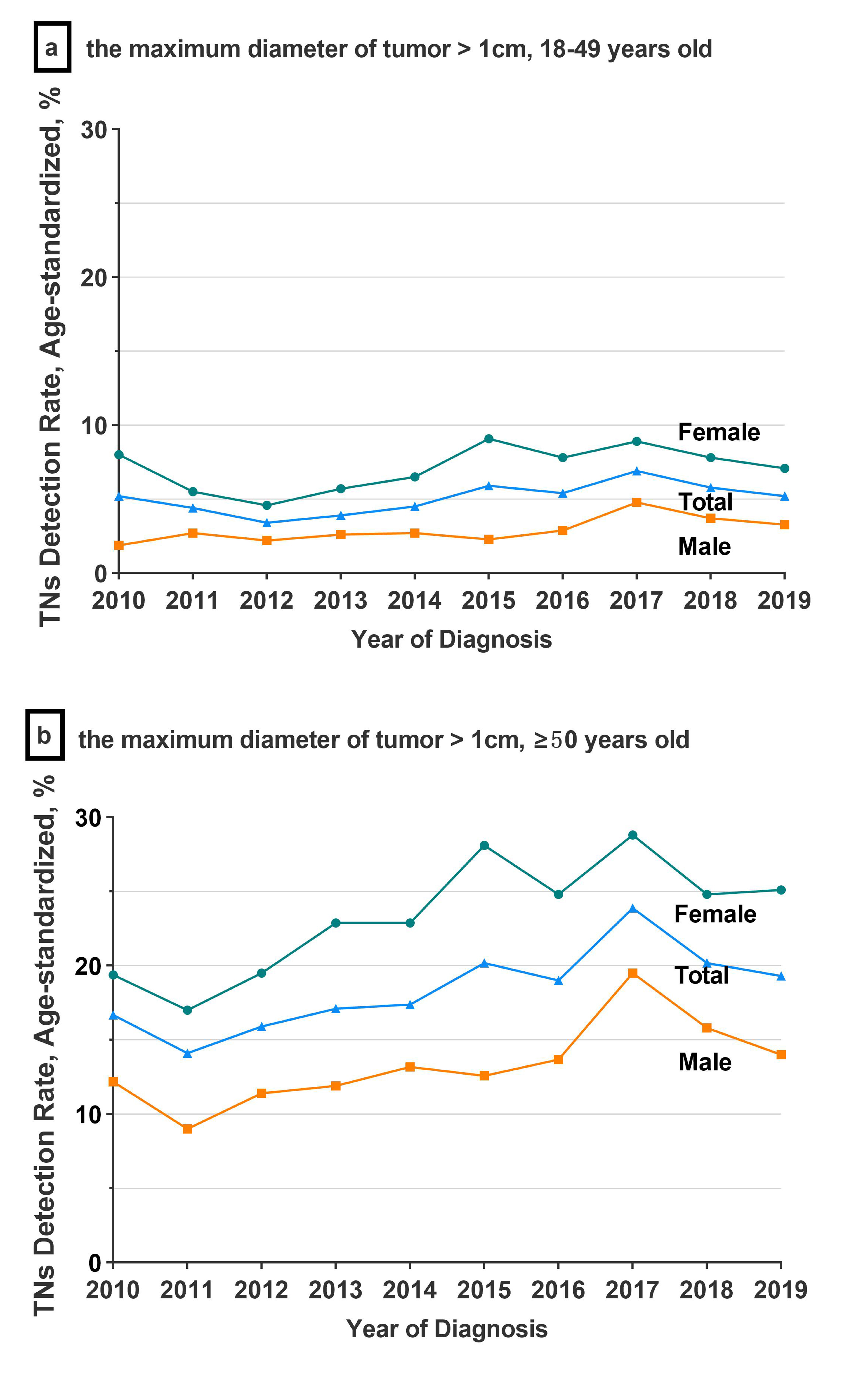


Supplemental Figure 4. Trends in Detection Rates of TNs with the maximum diameter ≤1cm, Stratified by age, 2010-2019


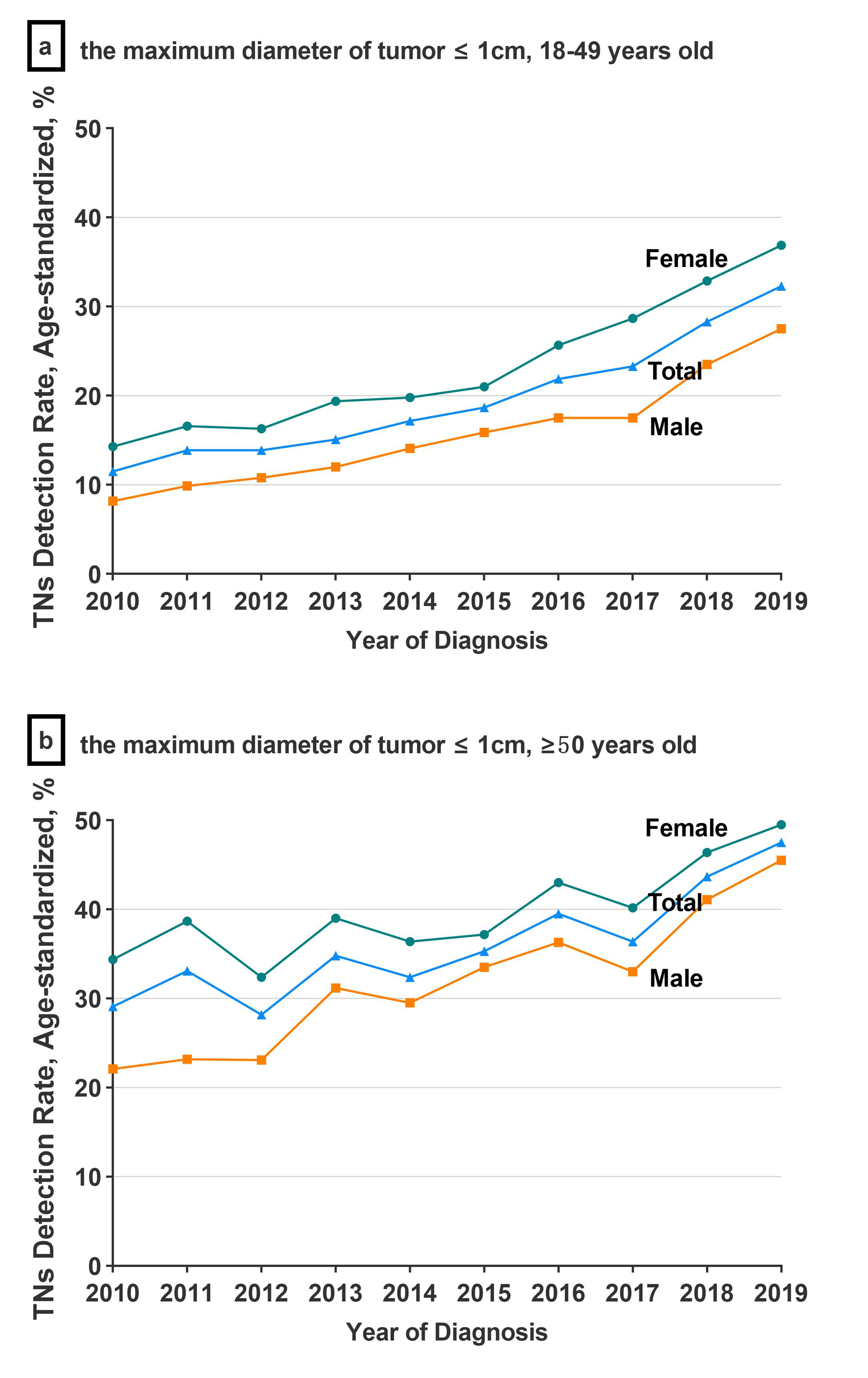


Supplemental Figure 5. Multivariable Regression Models for TSH Level with Restricted Cubic Splines.


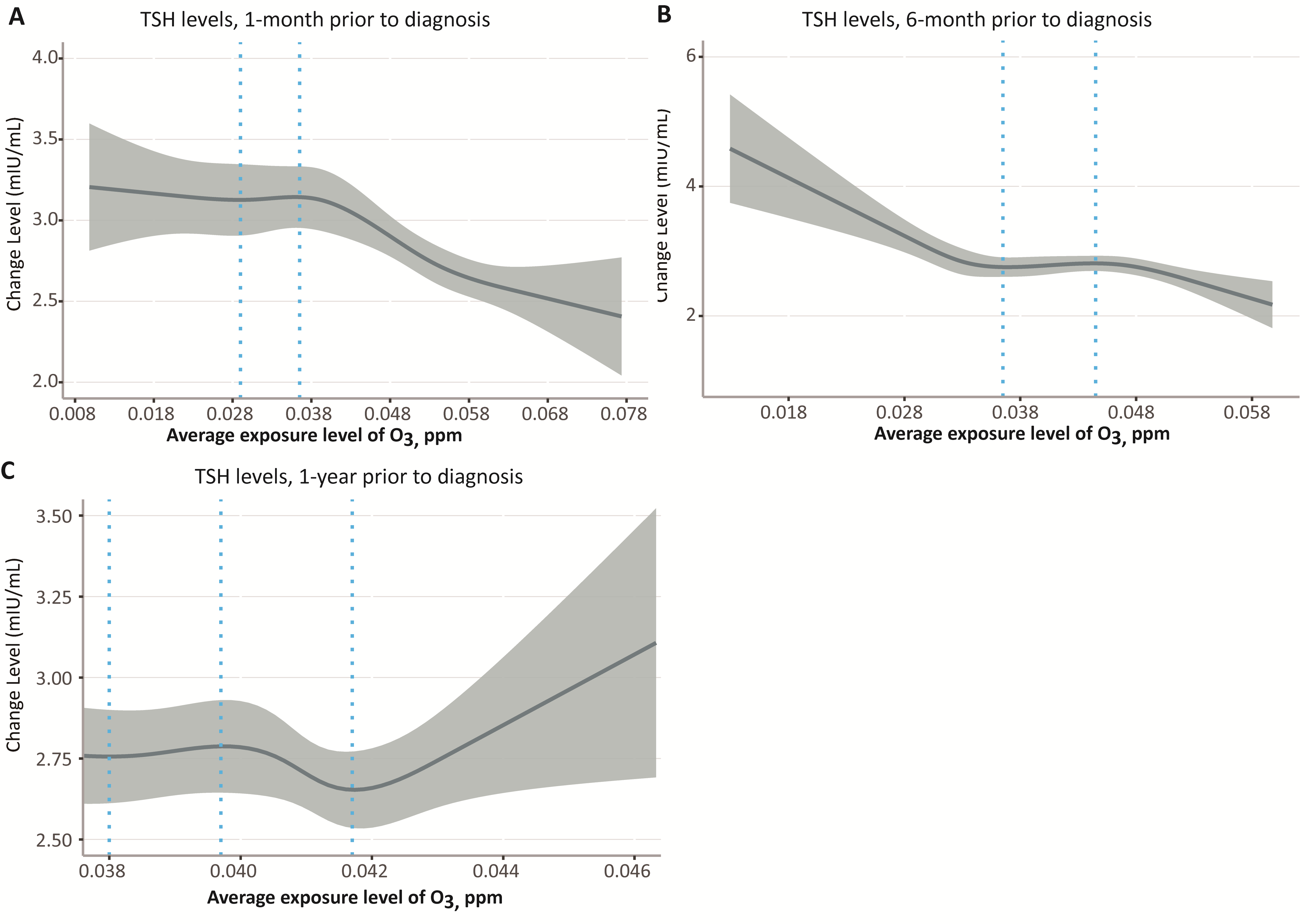

Supplement: Supplementary file 1 — Additional file 1: Supplemental Table 1. Basic characteristics of 39,353 patients with thyroid function. Supplemental Table 2. Age-standardized detection rates of TNs in Hunan Province from 2010 to 2019, stratified by tumor size, all age, both sexes. Supplemental Table 3. Age-standardized detection rates of TNs in Hunan Province from 2010 to 2019, stratified by age, all tumor sizes, both sexes. Supplemental Table 4. Age-standardized detection rates of TNs in Hunan Province from 2010 to 2019, stratified by sex, all tumor sizes, all ages. Supplemental Figure 1. Trends in TNs Detection Rates, Stratified by Sex, all ages, 2010–2019. Supplemental Figure 2. Trends in TNs Detection Rates, Stratified by age, all tumor sizes, 2010–2019. Supplemental Figure 3. Trends in Detection Rates of TNs with the maximum diameter > 1 cm, Stratified by age, 2010–2019. Supplemental Figure 4. Trends in Detection Rates of TNs with the maximum diameter ≤ 1 cm, Stratified by age, 2010–2019. Supplemental Figure 5. Multivariable Regression Models for TSH Level with Restricted Cubic Splines. [file 12940_2022_874_MOESM1_ESM.doc]
